# Evaluating the impact of altitudinal variations on COVID-19 mortality rates: a comprehensive analysis

**DOI:** 10.1186/s12890-025-03900-w

**Published:** 2025-08-30

**Authors:** Emre Karsli, Damla Anbarli Metin, Arda Kocatas, Feride Fulya Ercan, Ramazan Sabirli, Aylin Koseler, Matteo Pellegrini

**Affiliations:** 1 Faculty of Medicine Department of Emergency Medicine, Cigli Training and Research Hospital, Izmir Bakircay University, Izmir, Turkey; 2 Faculty of Medicine, Department of Emergency Medicine, Karabuk University, Karabuk, Turkey; 3 Faculty of Medicine, Department of Biophysics, Pamukkale University, Denizli, Turkey; 4 David Geffen School of Medicine, Department of Molecular, Cell & Developmental Biology, University of California Los Angeles, Los Angeles, USA

## Abstract

**Background:**

High altitude, known for its effects on respiratory diseases, was analyzed for its potential protective role. Data from the New York Times COVID-19 repository, U.S. Census Bureau, and topographic maps were utilized, covering January 2020 to August 2022, including pre- and post-vaccination periods. This study investigates the influence of high altitude on COVID-19 mortality, fatality rates, and vaccination outcomes in the United States. COVID-19, caused by SARS-CoV-2, has shown significant disparities in severity and outcomes across populations.

**Methods:**

This study utilized publicly available data from the New York Times COVID-19 repository and the US Census Bureau’s American Community Survey to analyze case fatality rates across mainland US counties from January 21, 2020, to August 13, 2022. Average altitude data were obtained from topographic maps, and counties outside the mainland USA were excluded. Vaccination-related data were assessed using the cutoff date of December 14, 2020. The rural-urban status of counties was determined using the Index of Relative Rurality (IRR) from Waldorf and Kim’s study, which included 3105 of 3109 counties.

**Results:**

Counties above 1500 m exhibited significantly lower case numbers, deaths, cases per million, and fatality rates compared to counties below this altitude. Pre-vaccination fatality rates were notably reduced in high-altitude regions (*p* = 0.0001), while post-vaccination data continued to demonstrate lower fatality rates (*p* < 0.0001). A positive correlation between rurality and post-vaccination fatality rates was observed (rho = 0.176, *p* = 0.0001). Altitude, alongside vaccination status, was identified as a critical factor influencing fatality rates (*p* = 0.001 for both). Additionally, a significant positive correlation between rurality (Index of Relative Rurality) and post-vaccination fatality rates was observed (rho = 0.176, *p* = 0.0001).

**Conclusions:**

Our findings highlight that high-altitude adaptations, such as increased lung capacity and epigenetic changes, may mitigate COVID-19 severity. However, the role of environmental and genetic factors remains insufficiently explored. Importantly, the study underscores healthcare inequities in rural high-altitude areas, where limited vaccination access exacerbates mortality risks. While altitude shows promise as a protective factor, addressing healthcare access disparities and further investigating high-altitude physiological and genetic adaptations are imperative for optimizing COVID-19 outcomes in diverse populations.

## Introduction

The recent global pandemic of Coronavirus Disease 2019 (COVID-19), attributable to the Severe Acute Respiratory Syndrome Coronavirus-2 (SARS-CoV-2), has led to a significant surge in cases of acute respiratory failure and Acute Respiratory Distress Syndrome (ARDS). These conditions are intrinsically linked to high mortality rates, rising healthcare expenditures, and increased post-recovery morbidity [1]. The severe progression and high fatality and morbidity rates of COVID-19, coupled with observed disparities in mortality outcomes, have driven researchers to investigate the underlying factors contributing to these variations.

The influence of altitude on respiratory diseases has been extensively addressed by prior literature. Previous investigations in this domain have noted that mortality rates associated with chronic lower respiratory tract diseases, pneumonia, and influenza tend to rise with high altitude [2, 3].

The differing severity of COVID-19 across various populations has prompted extensive research into its underlying causes. In that regard, the potential impact of high altitude on respiratory diseases has garnered significant interest, particularly in exploring the relationship between high altitude and COVID-19, a disease known for inducing severe respiratory distress.

Severe hypoxemia is among the most frequently observed complications in patients with COVID-19. Therefore, a significant number of these infected patients required intensive care, including not only supplemental oxygen but also non-invasive and invasive mechanical ventilation support. Moreover, even after receiving treatment for COVID-19 pneumonia, some patients continued to require oxygen therapy post-hospital discharge.

Numerous studies have established that hypoxia is independently linked to increased hospital mortality in COVID-19 patients [4]. Considering this association between COVID-19-related mortality and hypoxia, it is imperative to identify the impact of low oxygen pressure at high altitudes on patients afflicted with COVID-19.

Research addressing the interplay between the pathogenesis of COVID-19 and high altitude suggests that the severity of pathogenesis diminishes with increasing altitude [5]. Other studies also suggest that high altitude may serve as a protective factor against mortality from COVID-19 [6].

Prior work has found that while high altitude may lower COVID-19 infection rates, it does not confer a fully protective effect against the disease, nor does it reduce case-fatality rates [7]. By contrast, other studies indicate that individuals residing above 2000 m experience higher mortality rates from COVID-19 than their counterparts inhabiting below 1500 m [8].

Against this background, our study sought to investigate the association between high altitude and the incidence of COVID-19, vaccination rates, and mortality rates in the USA.

## Methods

### Study design and study population

The ethical approval of this epidemiological observational study was granted by the Ethics Committee of Izmir Bakircay (Numbered:1697–1717).

This study is based on the analysis of anonymized, aggregated mortality data obtained from official public records. No identifiable personal information was collected or used. As such, individual informed consent was not required according to the guidelines of our institutional review board and relevant ethical regulations. Altitude information for all mainland counties in the USA was obtained from topographic maps, and the average altitude data were recorded. Counties outside mainland USA were excluded from this study [9]. The counties were classified into two groups based on their altitude: high altitude (> 1500 m) and low altitude (< 1500 m [10].

Our analysis utilized data from the *New York Times*’ ongoing COVID-19 data repository to calculate case fatality rates (%) by US county from January 21, 2020, to August 13, 2022. COVID-19 data were integrated with the US *New York Times*’ ongoing COVID-19 data repository to Census Bureau’s American Community Survey. The dataset included the number of cases and deaths, fatality rates, case and death data per million people, vaccination data, and nationwide case and fatality rates [11, 12]. Moreover, the same data were recorded up to December 14, 2020, marking the inauguration of vaccinations in the USA. To assess the rural or urban characteristics of settlements, Index of Relative Rurality (IRR) data was incorporated, using IRR scores from Waldorf and Kim’s (2010) study that covered 3105 of 3109 counties [13].

### Statistical analysis

Statistical analyses were performed using the SPSS software program. Continuous variables were presented as medians and interquartile ranges (IQR). The normality of continuous data distributions was assessed using the Kolmogorov-Smirnov test. For comparing differences between independent groups. When the parametric test assumptions were not met, Mann-Whitney *U* test were performed for the comparison of independent group differences. Poisson regression analysis was performed to evaluate the associations of altitude, rurality (IRR), case incidence per million, and death per million with county-level COVID-19 fatality rates. Interaction terms (altitude × rurality, altitude × case per million, and altitude × death) were included to assess potential effect modification across pre- and post-vaccination periods. Additionally, the effects on fatality rates were analyzed using a Two-Way ANOVA. Statistical significance was defined as a p-value of less than 0.05.

## Results

A total of 185 out of 3109 mainland counties in the USA were identified to have altitudes exceeding 1500 m.

Analysis of data up to December 14, 2020, revealed that counties situated at > 1500 m had significantly lower numbers of cases, deaths, cases per million people and fatality rate compared to their < 1500 m counterparts (*p* = 0.000; *p* = 0.0001; *p* = 0.002;*p* = 0.0001 respectively) (Table 1).

**Table 1:** COVID-19case and death data before vaccination starts in USA (14th December 2020)

	< 1500 m Altitude (*N* = 2924)	> 1500 m Altitude (*N* = 185)	p-Value
Cases	1416 (600–3589)	685 (258–2637)	0.0001
Deaths	23 (9–59)	7 (2-26.5)	0.0001
Cases per million	14.9 (9.33–22.84)	12.35 (6.92–20.31)	0.002
Deaths per million	54250.7 (39360.3-71134.9)	52248.2 (35496.3-74423.9)	0.658
Fatality rate	15.15 (9.65–23.16)	8.62 (3.97–15.6)	0.0001

p-Values are derived from Mann-Whitney U test

For data up to August 13, 2022, counties at > 1500 m continued to exhibit significantly lower numbers of cases, deaths, cases per million, deaths per million, and fatality rates than counties at < 1500 m (*p* < 0.0001 for all variables) (Table 2).

**Table 2:** COVID-19case and death data now in USA

	< 1500 m Altitude (*N* = 2924)	> 1500 m Altitude (*N* = 185)	p-Value
Cases	7422.5 (3005–19654)	3710 (1554–15253)	**0.0001**
Deaths	107 (48–251)	37 (15.5-127.5)	**0.0001**
Cases per million	272541.5 (236767–307285)	255,422 (206323–295263)	**0.0001**
Deaths per million	3902 (2857.2–4925)	2265 (1547–3839)	**0.0001**
Fatality rate	13.92 (10.63–17.92)	11.26 (6.54–15.71)	**0.0001**

p-Values are derived from Mann-Whitney U test

**Fig. 1:**
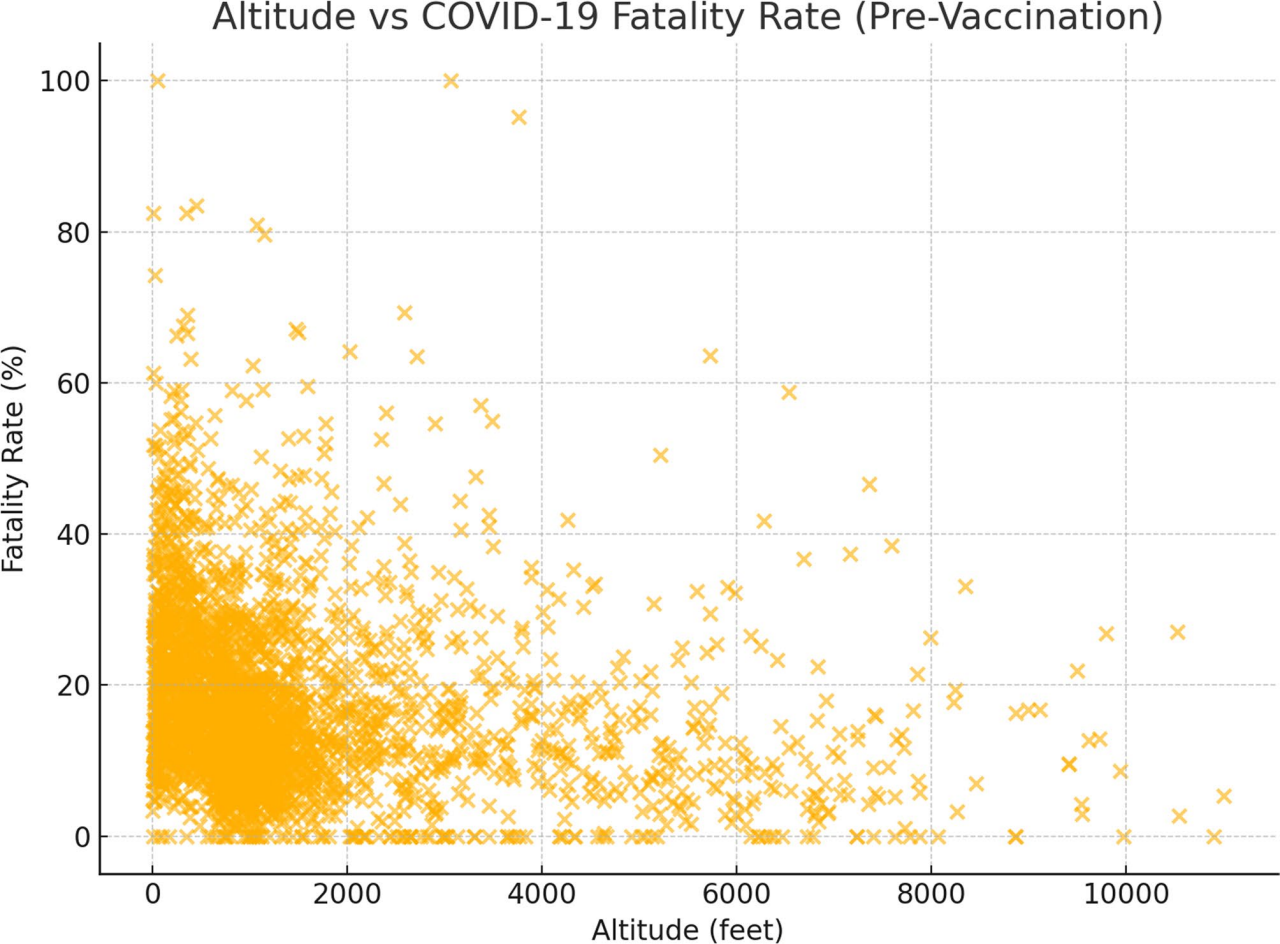
Association Between Altitude and COVID-19 Fatality Rate (Pre-Vaccination Period)

**Fig. 2:**
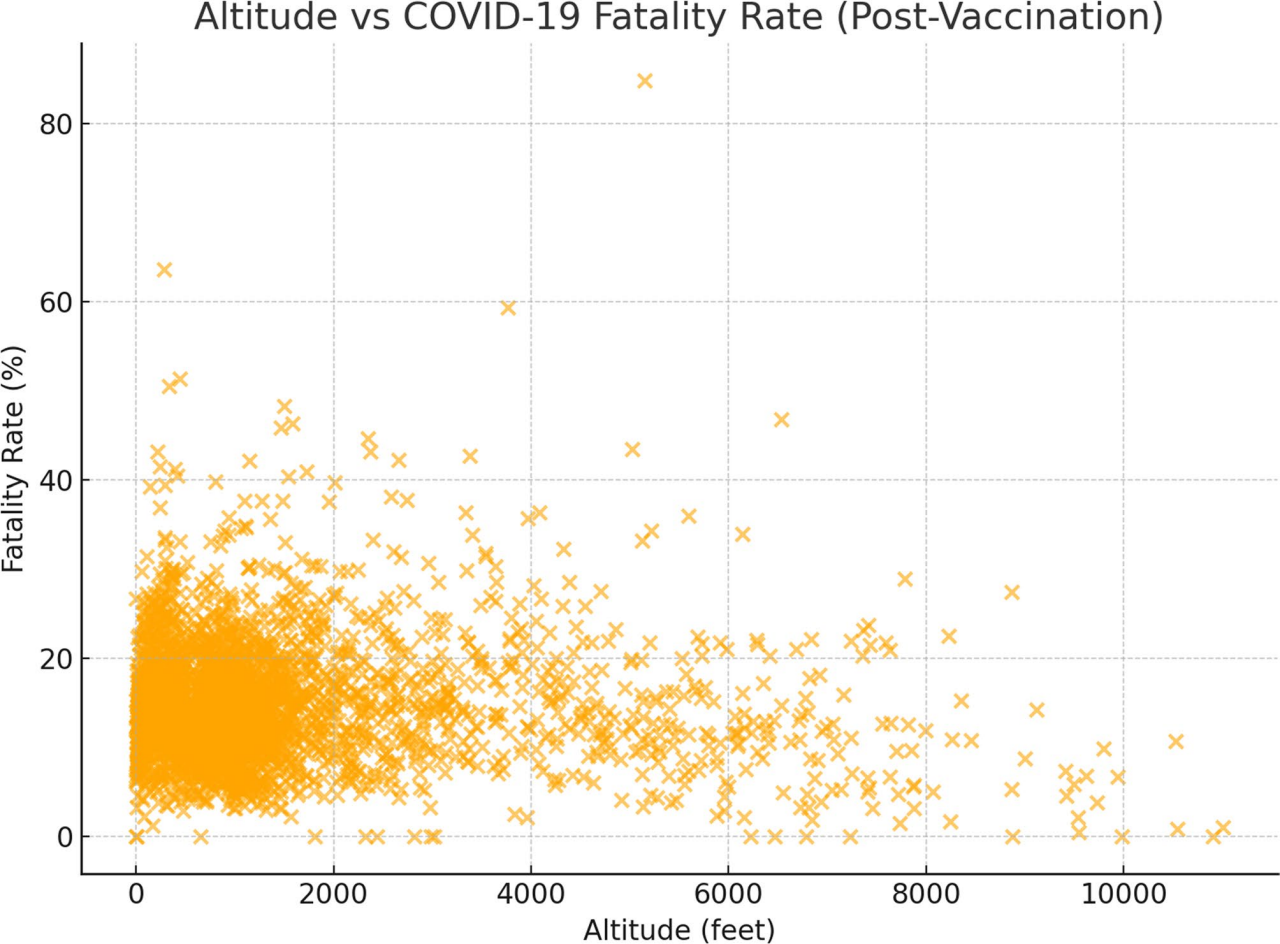
Association Between Altitude and COVID-19 Fatality Rate (Post-Vaccination Period)

Table 3 presents the correlational relationship between the IRR and various variables, such as pre- and post-vaccination case numbers, death numbers, vaccination rates, elevation, and fatality rates. No significant correlation was evident between IRR and pre-vaccination fatality rates (rho=−0.034, *p* = 0.059), whereas a significant positive correlation was observed with post-vaccination fatality rates (rho = 0.176, *p* = 0.0001). While no correlation was detected between altitude and pre-vaccination case and death numbers, a slight negative correlation was noted post-vaccination (Table 3).Scatter plots graphics show altitude and fatality relationships in pre-vaccination and post-vaccination periods (Figs. 1 and 2).

**Table 3:** Correlations between parameters

			Before Vaccination			After Vaccination					
			Cases, per million	Deaths, per million	Fatality rate	Cases, per million	Deaths, per million	At least one vaccination	Fully Vaccination	Fatality rate	Altitude
	IRR	rho	0.016	0.009	−0.034	−0.014	**0.153**	**−0.259**	**−0.0295**	**0.176**	**0.258**
		p Value	0.385	0.623	0.059	0.450	**0.001**	**0.001**	**0.001**	**0.0001**	**0.0001**
Before Vaccination	*Cases, * *per million *	rho		0.009	−0.030	**0.037**	0.002	−0.013	−0.020	−0.007	−0.004
		p Value		0.630	0.099	**0.040**	0.915	0.459	0.277	0.680	0.832
	*Deaths, per million *	rho			−0.012	0.016	0.004	−0.012	−0.026	0.003	−0.016
		p Value			0.513	0.371	0.826	0.511	0.152	0.853	0.381
	*Fatality rate*	rho				**−0.072**	**0.471**	0.034	−0.018	**0.511**	**−0.19**
		p Value				**0.001**	**0.001**	0.057	0.313	**0.0001**	**0.0001**
After Vaccination	*Cases, per million *	rho					**0.244**	**0.081**	0.001	**−0.196**	**−0.075**
		p Value					**0.001**	**0.001**	0.936	**0.0001**	**0.0001**
	*Deaths, per million *	rho						**−0.211**	**−0.345**	**0.823**	**−0.0128**
		p Value						**0.001**	**0.001**	**0.0001**	**0.0001**
	Fatality rate	rho						**−0.257**	**−0.347**		**−0.058**
		p Value						**0.0001**	**0.0001**		**0.001**

Bold *p* values indicate statistically significant results

Given the factors influencing fatality rates before the start of vaccination activities in the USA, altitude was found to exert a notable effect to reduce fatality rates (*p* = 0.0001). When it comes to the factors affecting current fatality rates, full vaccination status, at least one vaccination dose, and the county’s altitude prove to be significant determinants (*p* = 0.001; *p* = 0.002; *p* = 0.001, respectively). While the IRR score did not significantly affect pre-vaccination fatality rates (*p* = 0.352), it did show a substantial effect post-vaccination (*p* = 0.0001). Although vaccination status emerged as the most influential contributor, the average altitude of the county also exerted a moderate effect on fatality rates (Table 4).

**Table 4:** Covariance analysis

Tests of Between-Subjects Effects						
	***Source***	SS	***df***	MS	F	***p*** Value
***Before Vaccination***	IRR	119,589	1	119.589	0.866	0.352
	Altitude	15762.482	1	15762.482	114.106	0.0001
	Case per million	410.363	1	410.363	2.971	0.085
	Deaths per million	98.018	1	98.018	0.710	0.400
	Error	428231.475	3100	138,139		
	Total	1380265.804	3105			
	Corrected Total	444933.224	3104			
	**Source**	**SS**	***df***	**MS**	**F**	***p*** **Value**
***After Vaccination***	Fullv vaccined	6675.977	1	6675.977	172.398	0.001
	At least one vaccinated	380.167	1	380.167	9.817	0.002
	Altitude	870.304	1	870.304	22.474	0.001
	IRR	1305.615	1	1305.615	33.716	0.0001
	Error	120,044,765	3100	38.724		
	Total	807887.38	3105			
	Corrected Total	139167.654	3104			

R Squared = 0.137 (Adjusted R Squared = 0.136)

Dependent Variable: Fatality rate after vaccination

R Squared = 0 0.038 (Adjusted R Squared = 0.036)

Variable: Fatality rate before vaccination

**Fig. 3:**
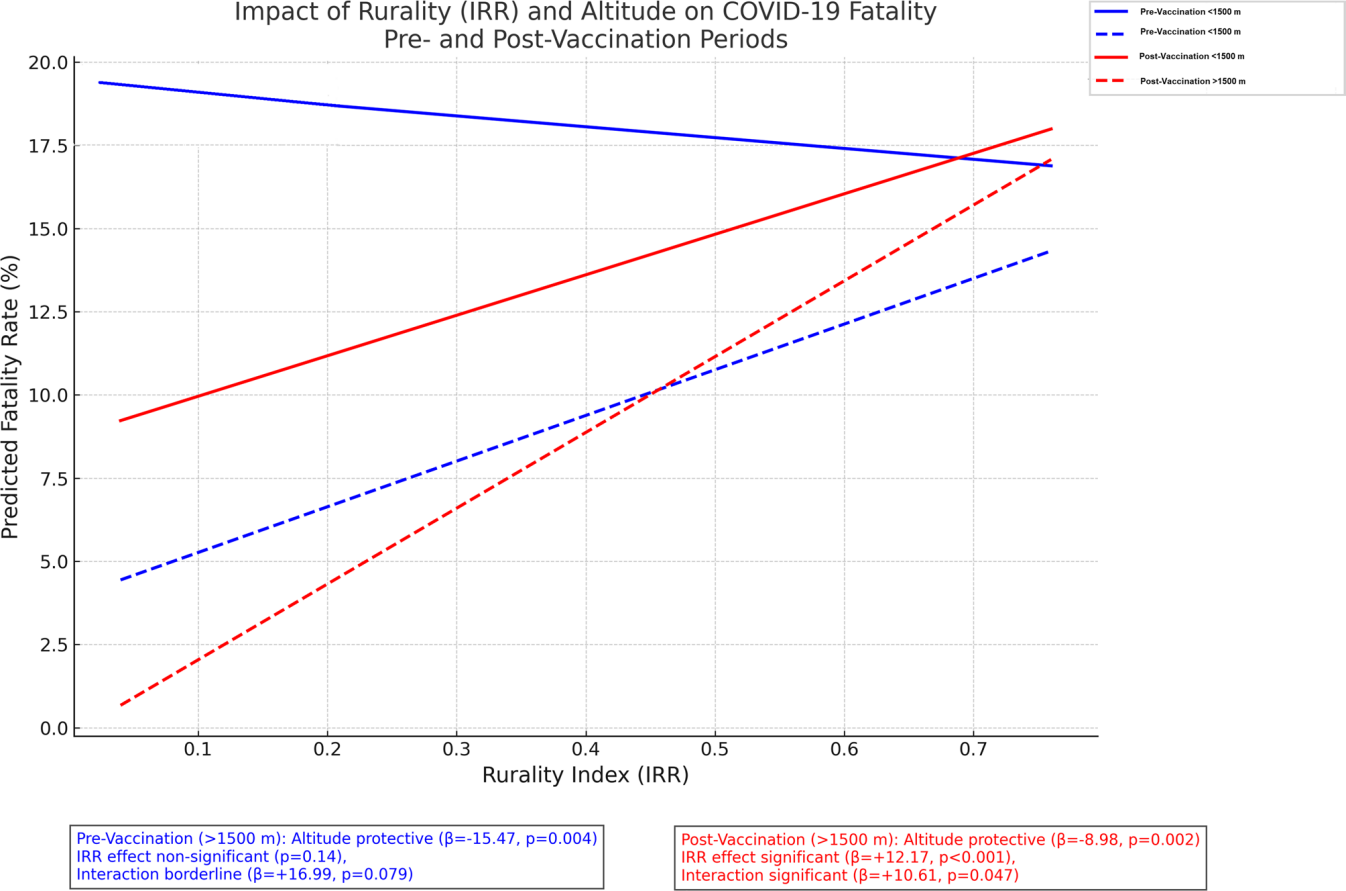
Impact of Rurality and Altitude on COVID-19 Fatality (Pre- and Post Vaccination Period)

In a multivariable linear regression model evaluating the pre-vaccination period, altitude (> 1500 m), rurality index (IRR), and their interaction were assessed as predictors of county-level fatality rates. The model demonstrated that residence at > 1500 m was independently associated with a significant reduction in fatality rates (β = − 15.47, 95% CI: − 25.98 to − 4.97, *p* = 0.004). Rurality alone was not a significant predictor (β = − 3.26, 95% CI: − 7.59 to 1.08, *p* = 0.141) (Fig. 3; Table 5).

However, the interaction term between altitude and rurality (IRR × Altitude) suggested a modifying effect (β = +16.99, 95% CI: − 1.96 to 35.94, *p* = 0.079). Specifically, while rurality was associated with a slight decrease in fatality at < 1500 m, in counties above 1500 m the effect reversed, with increasing rurality attenuating or even reversing the protective effect of altitude. The final model accounted for 1.7% of the variance in fatality rates (R² = 0.017). (Fig. 3; Table 5).

**Table 5:** Multivariable linear regression analysis of altitude and rurality index on COVID-19 fatality rates in the pre- and post-vaccination periods

Period	Predictor	β Coefficient	95% Confidence Interval	p-value	Adjusted R² (%)
Pre-vaccination	Altitude (>1500 m)	–15.47	–25.98 to –4.97	0.004	
	IRR	–3.26	–7.59 to 1.08	0.141	
	Altitude × Rurality	+16.99	–1.96 to 35.94	0.079	1.7
Post-vaccination	Altitude (>1500 m)	–8.98	–14.77 to –3.19	0.002	
	IRR	+12.17	9.78 to 14.56	<0.001	
	Altitude × Rurality	+10.61	0.16 to 21.06	0.047	4.5

IRR; Rurality index

In the post-vaccination period, a multivariable regression model including altitude (> 1500 m), rurality index (IRR), and their interaction as predictors of county-level fatality rates showed that rurality was a significant independent predictor (β = +12.17, 95% CI: 9.78–14.56, *p* < 0.001). Residence at > 1500 m remained protective (β = − 8.98, 95% CI: − 14.77 to − 3.19, *p* = 0.002). However, the interaction term (IRR × Altitude) was also significant (β = +10.61, 95% CI: 0.16–21.06, *p* = 0.047), indicating that the protective effect of altitude diminished and even reversed with increasing rurality. This model accounted for 4.5% of the variance in fatality rates (R² = 0.045) (Fig. 3; Table 5).

## Discussion

This study intended to explore the extent to which city altitude exerted an effect on COVID-19 mortality and fatality rates. As of December 14, 2020, coinciding with the onset of vaccination in the USA, we observed that counties situated at altitudes > 1500 m exhibited lower COVID-19 fatality rates in comparsion to the others below this altitude, though the deaths per million did not differ. Post-vaccination data, as of August 13, 2022, further revealed that both fatality rates and deaths per million remained lower in high-altitude counties.

Of all the US counties located above 1500 m, 3 are in Arizona, 6 in California, 49 in Colorado, 22 in Idaho, 18 in Montana, 1 in Nebraska, 15 in Nevada, 23 in New Mexico, 2 in Oregon, 29 in Utah and 17 in Wyoming. These high-altitude counties predominantly comprise rural and semi-urban areas [14].

**Fig. 4:**
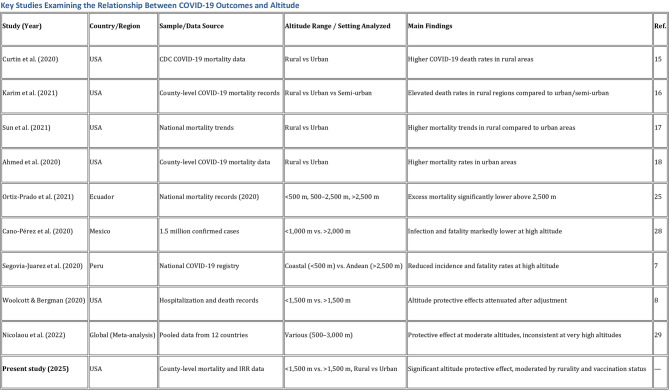
Key Studies Examining the Relationship Between COVID-19 Outcomes and Altitude

Reviewing literature on COVID-19 mortality across rural and urban settings, Curtin et al. (2020), as summarized in a CDC brief, found higher COVID-19 death rates in rural areas [15]. Similarly, Karim et al. [16] and Sun et al. [17] noted elevated death rates in rural regions compared to urban and semi-urban areas. By contrast, Ahmed et al. reported higher mortality in urban areas [18]. The literature on COVID-19 mortality in rural and urban areas primarily focuses on the pre-vaccination period, and our study likewise offers data pertaining to this timeframe. In this regard, our study aligns with these findings, indicating no significant effect of the IRR score on COVID-19 deaths before vaccination. Key studies about literatüre has given in Fig. 4.

Limited studies have investigated COVID-19 case fatality before vaccination. Pro et al. (2020) analyzed 2542 American counties, revealing lower fatality rates in urban areas [19]. Our findings corroborate this, showing no significant effect of the IRR score on fatality rates in the pre-vaccination period, but post-vaccination data did indicate an impact of increased rurality on fatality rates.

As identified by multiple studies, residents of rural areas are likely to receive less healthcare services compared to those in urban regions [20–22]. Globally, access to vaccination is also more challenging in rural areas. Factors as diverse as climatic conditions, level of economic development, and investment in health amenities have been identified as significant determinants in studies exploring the drivers influencing COVID-19 fatality [23].

Given that the fatality rate in our study is influenced by vaccination levels in both pre- and post- vaccination periods, we note that access to vaccination proved to be more challenging in rural areas. Confirming this, Saelee et al., who analyzed similar timeframes, reported that vaccination rates remained higher in urban areas than in rural regions [24]. The contrast observed in the association between altitude and COVID-19 case and mortality metrics before and after the deployment of vaccination campaigns suggests the presence of a shift in epidemiological dynamics over the course of the period. In the early phase of the pandemic, these associations were weak or nonsignificant from a statistical point of view, possibly expressing a more homogeneous susceptibility among zones in the absence of widespread interventions. However, as vaccine campaigns persisted, associations became more robust, a likely reflection of variations in vaccine coverage, healthcare access, and public health infrastructure—conditions that are often defined by rurality and altitude. Rural and high-altitude counties may have experienced logistical obstacles in vaccine distribution and healthcare delivery, which could have enhanced the role of altitude-related factors on COVID-19 results. Such findings stress the importance of including sociodemographic context in accounts of geographical differences in pandemic impact. As further evidence for this position, Saelee et al. demonstrated that vaccination was consistently lower in rural U.S. counties, emphasizing the suggestion that disparities in access drove these post-vaccination mortality shifts [24].

When examining studies on COVID-19 mortality at different altitudes, irrespective of rural or urban settings, a 2021 study performed in Ecuador by Ortiz-Prado et al. found that COVID-19 mortality rates were lower in high and very high altitude cantons than in low altitude ones [25]. In addition, Jibaja et al. observed that patients treated at high altitudes in Ecuador were 74% more likely to survive ICU and 35% more likely to survive hospitalization than those treated at sea level [26]. In Saudi Arabia, Abdelsalam et al. discovered that inhabitants residing at higher altitudes developed less severe forms of COVID-19 and had lower mortality rates compared to those at sea level, through an as-yet unknown mechanism [27]. The existing COVID-19 literature also reported mixed results in relation to the disease and altitude. For instance, Segovia-Juarez et al. observed that while high altitude reduces infection rates, it does not impact the case-fatality rate [7]. Cano-Perez et al. noted that residence at high altitude decreases the COVID-19 case fatality rate, whereas Nicolaou et al. found no reduction in COVID-19 mortality risk associated with high altitude [28, 29]. Our study identified the impact of altitude upon COVID-19 case fatality rates in both pre- and post-vaccination periods, aligning with findings which suggest that high altitude mitigates COVID-19 case fatality. Although rurality did not significantly influence COVID-19 case fatality prior to vaccination and produced a negative effect post-vaccination, we infer that high-altitude residence has a more substantial impact on COVID-19 case fatality than rural living.

While the mechanisms underlying reduced COVID-19 fatality at high altitudes remain unclear, chronic hypoxemia, climatic variations, air pollution, environmental, and epigenetic factors are hypothesized to account for this relationship. In individuals residing at high altitudes, total lung capacity and vital capacity progressively increase over time. Membrane diffusion capacity in the lungs also rises due to elevated hemoglobin levels and increased alveolar volume [30–33]. Apart from these elevations in lung capacity and function, chronic hypoxia induces epigenetic changes, facilitating adaptation to environmental conditions [34]. The role of various genetic and epigenetic factors, particularly the hypoxia-inducible factor pathway and reduced Angiotensin Converting Enzyme-2 (ACE2) gene expression, is also well-documented [5, 35–40].

Our findings show that the relationship between elevation and COVID-19 death is conditional on the underlying sociodemographic context, or rurality. Even though higher elevation appears to have a protective influence overall, that benefit is not equally present in every situation. In more rural settings, especially where elevation is higher, mortality rates were worse compared to urban ones. This pattern may account for variations in healthcare access, emergency response capacity, and vaccine distribution in mountain and rural areas.

Literature has already shown that high-altitude environments may offer physiological advantages, including greater oxygenation through long-term hypoxic adaptation [41]. However, these biological advantages may be weakened by rural-based structural disadvantages. In particular, rural U.S. county vaccination rates have consistently been lower than in metropolitan areas, as shown by Saelee et al., and are likely to be exacerbating regional differences in pandemic outcomes [24]. Such complexities have been recognized before by Woolcott and Bergman, who reported that mortality at high altitudes varied depending on sociodemographic and healthcare characteristics [8].

These findings underscore the importance of both environmental and structural determinants in explaining spatial variation in COVID-19 severity. Altitude will probably play some contributory effect, but its impact appears to be conditioned by broader public health inequalities.

Although we did not specifically investigate genetic or environmental factors, our findings imply that these adaptations could contribute to the reduced fatality rates observed at high altitudes. The slight positive correlation between altitude and IRR score, coupled with the negligible effect of the IRR score on pre-vaccination fatality, highlights the significance of high-altitude adaptations and environmental influences on COVID-19 outcomes.

Our study demonstrated significantly lower COVID-19 case fatality rates in U.S. counties located above 1500 m. This association may not solely be explained by environmental exposure but may also involve physiological adaptations to chronic hypoxia. In high-altitude populations, angiogenesis and increased pulmonary capillarization are known to enhance oxygen transport under hypoxemic conditions and may contribute to improved survival in respiratory illnesses such as COVID-19 [36, 41, 42].

However, recent studies have shown no significant difference in SARS-CoV-2 viral loads by altitude, suggesting that transmission dynamics, including population density, human mobility, vaccination coverage, and healthcare accessibility, may play equally or more influential roles [19, 28, 43].

Importantly, our analysis is based exclusively on U.S. county-level data, which primarily involve populations residing at moderate altitudes and lacking long-term genetic or epigenetic adaptations observed in highland regions such as the Andes, Himalayas, or Ethiopian Plateau [8, 44, 45].

Therefore, while our results suggest a protective association between altitude and COVID-19 fatality within the U.S. context, these findings should be interpreted cautiously and not generalized globally without accounting for distinct genetic backgrounds, environmental exposures, and sociodemographic differences.

One limitation of our study is the absence of excess mortality metrics. While excess mortality is considered a more accurate indicator of the pandemic’s true impact, especially in regions with limited testing capacity or underdeveloped reporting systems, standardized and comparable county-level excess mortality data were not available for the continental United States across both the pre- and post-vaccination periods. As a result, our reliance on reported deaths and case fatality rates may have underestimated the actual burden of COVID-19 in certain areas.

Another limitation of this study is its reliance exclusively on U.S. county-level data, which represent relatively modest elevations and populations with limited long-term physiological adaptation to high altitude compared to regions such as the Andes, Himalayas, or Ethiopian Highlands. Therefore, our findings should be interpreted cautiously when extrapolating to populations residing at higher elevations with distinct adaptive characteristics.

## Conclusions

Overall, our study demonstrates that residence at high altitudes is associated with reducing COVID-19 case fatality and mortality rates, which can be attributed to environmental factors and physiological and epigenetic adaptations induced by high altitude.

COVID-19 case fatality impacts society as a whole, transcending rural or urban distinctions. Our findings highlight the indirect effects of healthcare access disparities and vaccination challenges in rural areas, as evidenced by the impact of the IRR score on post-vaccination fatality rates.

Considering that COVID-19 fatality rates in high-altitude and urban areas are likely unaffected by rurality and benefit from high-altitude adaptation mechanisms, we predict significantly lower fatality rates in these regions.

Our study is distinguished from existing literature by examining the effects on fatality and mortality data both pre- and past-vaccination, thus adding to the generality of our results. However, an important limitation of our study is the lack of examination of epigenetic and environmental factors. Epigenetic changes in high-altitude residents represent a critical area for further scientific investigation. Future studies analyzing environmental pollution and climate conditions will contribute significantly to the literature on this topic. Furthermore, recognizing the diverse demographic structures of different settlements, the absence of sociodemographic data is another limitation of our study.

## Data Availability

The data used in this study were derived from The New York Times’ COVID-19 Data Repository, which provides publicly available, continuously updated information on COVID-19 cases and deaths in the United States. The dataset includes cumulative and daily confirmed cases and deaths at both state and county levels.The repository is maintained on GitHub and can be accessed at: [https://github.com/nytimes/covid-19-data](https:/github.com/nytimes/covid-19-data)Additional daily surveillance data by state were sourced from the Centers for Disease Control and Prevention (CDC). Demographic and socioeconomic data used for population-level analysis were derived from the U.S. Census Bureau’s American Community Survey (ACS) and downloaded in CSV format via their public data portal. The dataset can be accessed at: [https://data.cdc.gov](https:/data.cdc.gov) or directly via [https://data.cdc.gov/Case-Surveillance/United-States-COVID-19-Cases-and-Deaths-by-State-o/9mfq-cb36](https:/data.cdc.gov/Case-Surveillance/United-States-COVID-19-Cases-and-Deaths-by-State-o/9mfq-cb36)And also CSV file is publicly available on Zenodo at the following DOI: [https://doi.org/10.5281/zenodo.15265480](https:/doi.org/10.5281/zenodo.15265480). To ensure long-term accessibility, the dataset has also been archived using a persistent tokenized link [46].

## Abbreviations

COVID-19: Coronavirus Disease 2019
SARS-CoV-2: Severe Acute Respiratory Syndrome Coronavirus-2
ARDS: Acute Respiratory Distress Syndrome
IRR: Index of Relative Rurality
CDC: Centers of Disease Control and Prevention
ACE2: Angiotensin Converting Enzyme

## References

[1] Cesta MC, Zippoli M, Marsiglia C, et al. The role of interleukin-8 in lung inflammation and injury: implications for the management of COVID-19 and hyperinflammatory acute respiratory distress syndrome. Front Pharmacol. 2022. 10.3389/fphar.2021.808797. 35095519 10.3389/fphar.2021.808797 PMC8790527

[2] Pérez-Padilla R, Franco-Marina F. The impact of altitude on mortality from tuberculosis and pneumonia. Int J Tuberc Lung Dis. 2004;8:1315–20. 15581198

[3] Hwang J, Jang M, Kim N, et al. Positive association between moderate altitude and chronic lower respiratory disease mortality in united States counties. PLoS One. 2018;13:e0200557. 10.1371/journal.pone.0200557. 29995931 10.1371/journal.pone.0200557 PMC6040762

[4] Xie J, Covassin N, Fan Z, et al. Association between hypoxemia and mortality in patients with COVID-19. Mayo Clin Proc. 2020;95:1138–47. 32376101 10.1016/j.mayocp.2020.04.006 PMC7151468

[5] Arias-Reyes C, Zubieta-DeUrioste N, Poma-Machicao L, et al. Does the pathogenesis of SARS-CoV-2 virus decrease at high-altitude? Respir Physiol Neurobiol. 2020;277: 103443. 10.1016/j.resp.2020.103443. 32333993 10.1016/j.resp.2020.103443 PMC7175867

[6] Thomson TM, Casas F, Guerrero HA, et al. Potential protective effect from COVID-19 conferred by altitude: a longitudinal analysis in Peru during full lockdown. High Alt Med Biol. 2021;22(2):209–24. 10.1089/ham.2020.0202. 33780636 10.1089/ham.2020.0202

[7] Segovia-Juarez J, Castagnetto JM, Gonzales GF. High altitude reduces infection rate of COVID-19 but not case-fatality rate. Respir Physiol Neurobiol. 2020;281: 103494. 10.1016/j.resp.2020.103494. 32679369 10.1016/j.resp.2020.103494 PMC7361094

[8] Woolcott OO, Bergman RN. Mortality attributed to COVID-19 in high-altitude populations. High Alt Med Biol. 2020;21(4):409–16. 10.1089/ham.2020.0098. 32815745 10.1089/ham.2020.0098

[9] Topographic Map. https://en-gb.topographic-map.com/ (Accessed 13 Aug 2022).

[10] Mathew TM, Sharma S. High Altitude Oxygenation. https://www.ncbi.nlm.nih.gov/books/NBK539701/. 30969523

[11] COVID-19 data. https://github.com/nytimes/covid-19-data (Accessed 13 Aug 2022).

[12] United States Census Bureau. American Community Survey data. https://data.cdc.gov/Case-Surveillance/United-States-COVID-19-Cases-and-Deaths-by-State-o/9mfq-cb36 (Accessed 13 Aug 2020).

[13] Waldorf B, Kim A. The index of relative rurality (IRR): US county data for 2000 and 2010. Purdue Univ Res Repository. 2018. 10.4231/R7959FS8.

[14] Rural Health Information Hub. https://www.ruralhealthinfo.org/data-explorer?classification=Metropolitan (Accessed 13 Aug 2022).

[15] Centers for Disease Control and Prevention. COVID-19 Death Rates in Urban and Rural Areas: United States 2020. https://www.cdc.gov/nchs/products/databriefs/db447.htm (Accessed 13 Aug 2022).

[16] Karim SA, Chen HF, Deaths From. COVID-19 in rural, micropolitan, and metropolitan areas: A County-Level comparison. J Rural Health. 2021;37:124–32. 10.1111/jrh.12533. 33155723 10.1111/jrh.12533

[17] Sun Y, Cheng K, Monnat S. Rural-Urban and Within-Rural differences in COVID-19 mortality trends. 2021. 10.31235/osf.io/jbhvs

[18] Ahmed R, Williamson M, Hamid M, et al. United States county-level COVID-19 death rates and case fatality rates vary by region and urban status. Healthcare. 2020;8(3): 330. 10.3390/healthcare8030330. 32917009 10.3390/healthcare8030330 PMC7551952

[19] Pro G, Hubach R, Wheeler D, et al. Differences in US COVID-19 case rates and case fatality rates across the urban-rural continuum. Rural Remote Health. 2020;20:6074. 10.22605/rrh6074. 32811154 10.22605/RRH6074 PMC7583661

[20] Chen X, Orom H, Hay J, et al. Differences in rural and urban health information access and use. J Rural Health. 2018;34:314–22. 10.1111/jrh.12335. 30444935 10.1111/jrh.12335 PMC6522336

[21] Reilly M. Health disparities and access to healthcare in rural vs. urban areas. Theory Action. 2021;14:9–30. 10.3798/TIA.1937-0237.2109.

[22] Arruda N, Maia A, Alves L. Inequality in access to health services between urban and rural areas in brazil: A disaggregation of factors from 1998 to 2008. Cad Saude Publica. 2018;34:e00213816. 10.1590/0102-311X00213816. 29947662 10.1590/0102-311X00213816

[23] Li M, Zhang Z, Cao W, et al. Identifying novel factors associated with COVID-19 transmission and fatality using the machine learning approach. Sci Total Environ. 2021;764: 142810. 10.1016/j.scitotenv.2020.142810. 33097268 10.1016/j.scitotenv.2020.142810 PMC7550892

[24] Saelee R, Zell E, Murthy B, et al. Disparities in COVID-19 vaccination coverage between urban and rural counties — United states, December 14, 2020–January 31, 2022. MMWR Morb Mortal Wkly Rep. 2022;71:335–40. 10.15585/mmwr.mm7109a2. 35239636 10.15585/mmwr.mm7109a2 PMC8893338

[25] Ortiz-Prado E, Naranjo R, Vasconez E, et al. Analysis of excess mortality data at different altitudes during the COVID-19 outbreak in Ecuador. High Alt Med Biol. 2021;22:406–16. 10.1089/ham.2021.0070. 34905395 10.1089/ham.2021.0070

[26] Jibaja M, Roldan-Vasquez E, Rello J, et al. Effect of high altitude on the survival of COVID-19 patients in intensive care unit: a cohort study. J Intensive Care Med. 2022;37:1265–73. 10.1177/08850666221099827. 35532089 10.1177/08850666221099827 PMC9095997

[27] Abdelsalam M, Althaqafi R, Assiri S, et al. Clinical and laboratory findings of COVID-19 in high-altitude inhabitants of Saudi Arabia. Front Med. 2021. 10.3389/fmed.2021.670195. 10.3389/fmed.2021.670195 PMC8149591 34055842

[28] Cano-Pérez E, Torres-Pacheco J, Fragozo-Ramos M, et al. Negative correlation between altitude and COVID-19 pandemic in Colombia: a preliminary report. Am J Trop Med Hyg. 2020;103:2347–9. 10.4269/ajtmh.20-1027. 33124543 10.4269/ajtmh.20-1027 PMC7695107

[29] Nicolaou L, Steinberg A, Carrillo-Larco R Living at high altitude and COVID-19 mortality in Peru. High Alt Med Biol 2022., Droma T, McCullough R, Zhuang J et al. (1991) Increased vital and total lung capacities in Tibetan compared to Han residents of Lhasa (3658 m). Am J Phys Anthropol 86:341–351. 10.1002/AJPA.1330860303. 10.1002/ajpa.1330860303 1746642

[30] Droma T, McCullough R, Zhuang J, et al. Increased vital and total lung capacities in Tibetan compared to Han residents of Lhasa (3658 m). Am J Phys Anthropol. 1991;86:341–51. 10.1002/AJPA.1330860303. 1746642 10.1002/ajpa.1330860303

[31] Basagaña X. Household air pollution as an important factor in the complex relationship between altitude and COPD. Eur Respir J. 2019. 10.1183/13993003.02454-2018. 30759422 10.1183/13993003.02454-2018

[32] Weitz C, Garruto R, Chin C, et al. Lung function of Han Chinese born and Raised near sea level and at high altitude in Western China. Am J Hum Biol. 2002;14:111–22. 10.1002/ajhb.10063. 10.1002/ajhb.10063 12112571

[33] Agostoni P, Swenson E, Bussotti M, et al. High-altitude exposure of three weeks duration increases lung diffusing capacity in humans. J Appl Physiol. 2011;110:1564–71. 10.1152/japplphysiol.01167.2010. 21436463 10.1152/japplphysiol.01167.2010

[34] Julian C. An aptitude for altitude: are epigenomic processes involved? Front Physiol. 2019;10: 1397. 10.3389/fphys.2019.01397. 31824328 10.3389/fphys.2019.01397 PMC6883803

[35] Childebayeva A, Jones T, Goodrich J, et al. LINE-1 and EPAS1 DNA methylation associations with high-altitude exposure. Epigenetics. 2019;14:1–15. 10.1080/15592294.2018.1561117. 30574831 10.1080/15592294.2018.1561117 PMC6380404

[36] Bigham A, Lee F. Human high-altitude adaptation: forward genetics meets the HIF pathway. Genes Dev. 2014;28:2189–204. 10.1101/gad.250167.114. 25319824 10.1101/gad.250167.114 PMC4201282

[37] Bigham A. Genetics of human origin and evolution: high-altitude adaptations. Curr Opin Genet Dev. 2016;41:8–13. 10.1016/j.gde.2016.06.018. 27501156 10.1016/j.gde.2016.06.018 PMC5161537

[38] Lorenzo F, Huff C, Myllymäki M, et al. A genetic mechanism for Tibetan high-altitude adaptation. Nat Genet. 2014;46:951–6. 10.1038/ng.3067. 25129147 10.1038/ng.3067 PMC4473257

[39] Dang Z, Su S, Jin G, et al. Tsantan sumtang attenuated chronic hypoxia-induced right ventricular structure remodeling and fibrosis by equilibrating local ACE-AngII-AT1R/ACE2-Ang1-7-Mas axis in rat. J Ethnopharmacol. 2020;250: 112470. 10.1016/j.jep.2019.112470. 31862407 10.1016/j.jep.2019.112470

[40] Zhang R, Wu Y, Zhao M, et al. Role of HIF-1α in the regulation ACE and ACE2 expression in hypoxic human pulmonary artery smooth muscle cells. Am J Physiol Lung Cell Mol Physiol. 2009;297:L631–40. 10.1152/ajplung.90415.2008. 19592460 10.1152/ajplung.90415.2008

[41] Simbaña-Rivera K, Jaramillo PRM, Silva JVV, et al. High-altitude is associated with better short-term survival in critically ill COVID-19 patients admitted to the ICU. PLoS One. 2022;17(3):e0262423. 10.1371/journal.pone.0262423. (**PMID: 35358185; PMCID: PMC8970356.b**). 35358185 10.1371/journal.pone.0262423 PMC8970356

[42] Julian CG. An aptitude for altitude: are epigenomic processes involved? Front Physiol. 2019;10:1397. 10.3389/fphys.2019.01397. 31824328 10.3389/fphys.2019.01397 PMC6883803

[43] Ortiz-Prado E, Simbaña-Rivera K, Gómez-Barreno L, et al. SARS-CoV-2 viral load analysis at low and high altitude: A case study from Ecuador. Viruses. 2022;14(2):329. 10.3390/v14020329. 35805606 10.3390/ijerph19137945 PMC9265329

[44] Beall CM. Two routes to functional adaptation: Tibetan and Andean high-altitude natives. Proc Natl Acad Sci U S A. 2007;104(Suppl 1):8655–60. 10.1073/pnas.0701985104. 17494744 10.1073/pnas.0701985104 PMC1876443

[45] Childebayeva A, Jones TL, Goodrich JM, et al. LINE-1 and EPAS1 DNA methylation associations with high-altitude exposure. Epigenetics. 2019;14(1):1–15. 10.1080/15592294.2018.1561117. 30574831 10.1080/15592294.2018.1561117 PMC6380404

[46] Epidemiology, COVID-19. CVS file available at: https://zenodo.org/records/15265480?token=eyJhbGciOiJIUzUxMiJ9.eyJpZCI6ImU4OTM0ZWFkLTQzYTUtNDY0Mi1hYjdlLWU0YTk2Yjg3MTU4YSIsImRhdGEiOnt9LCJyYW5kb20iOiIzMGM5MTc5ZTAzMGEzODdkZGZkZmIyYTQ1ZTIwODFjOCJ9.o7KTmwxXxMrxnb0zOJq5cQdMx1SgyWsgDNkdfB8KH8lidWeDqJeIBuyCKptvu28w740BQePhmlb5RVP0Efdk8w.

